# Sebaceous Tumors

**Published:** 1877-07

**Authors:** 


BILLROTH has gone to Kischenew, Roumania, the base of Russian military operations. His mission is supposed to be connected with the general hospital service of the Czar's immense army.

---

SEBACEOUS TUMORS.—Says the *Medical and Surgical Reporter*, Dr. B. Hamilton injects sebaceous tumors with the strong tincture of iodine, after evacuating the contents by a small puncture. No unpleasant effects result, and no scar remains from the puncture.

